# Electrospun Poly(lactide) Fibers as Carriers for Controlled Release of Biochanin A

**DOI:** 10.3390/pharmaceutics14030528

**Published:** 2022-02-27

**Authors:** Ivana Gajić, Sanja Stojanović, Ivan Ristić, Snežana Ilić-Stojanović, Branka Pilić, Aleksandra Nešić, Stevo Najman, Ana Dinić, Ljiljana Stanojević, Maja Urošević, Vesna Nikolić, Ljubiša Nikolić

**Affiliations:** 1 Faculty of Technology, University of Niš, Bulevar oslobodjenja 124, 16000 Leskovac, Serbia; ivana@tf.ni.ac.rs (I.G.); anatacic@tf.ni.ac.rs (A.D.); stanojevic@tf.ni.ac.rs (L.S.); maja@tf.ni.ac.rs (M.U.); nikolicvesna@tf.ni.ac.rs (V.N.); nljubisa@tf.ni.ac.rs (L.N.); 2 Department of Biology and Human Genetics, Faculty of Medicine, University of Niš, Blvd. Dr Zorana Djindjica 81, 18108 Niš, Serbia; sanja.stojanovic@medfak.ni.ac.rs (S.S.); stevo.najman@medfak.ni.ac.rs (S.N.); 3 Department for Cell and Tissue Engineering, Faculty of Medicine, University of Niš, Blvd. Dr Zorana Djindjica 81, 18108 Niš, Serbia; 4 Faculty of Technology Novi Sad, University of Novi Sad, 18000 Novi Sad, Serbia; ivan.ristic@uns.ac.rs (I.R.); brapi@uns.ac.rs (B.P.); alexm@uns.ac.rs (A.N.)

**Keywords:** drug delivery, carriers, phytoestrogens, biochanin A, electrospinning, polylactide

## Abstract

The aim of this study is to investigate the possibility of using electrospun polylactide (PLA) fibers as a carrier of the phytoestrogen biochanin A. Polylactide fibers were prepared with different contents of biochanin A by using an electrospinning method at specific process parameters. The obtained electrospun polylactide fibers, as carriers of biochanin A, were characterized by means of different methods. The presented results showed that the mechanical properties of PLA have not changed significantly in the presence of biochanin A. Scanning electron microscopy showed that the fine fiber structure is retained without visible deformations and biochanin A crystals on the surface of the fibres. The analysis by infrared spectroscopy showed that there are no strong interactions between polylactide and biochanin A molecules, which is a good prerequisite for the diffusion release of biochanin A from PLA fibers.The release of biochanin A from PLA fibers in buffer solution pH 7.4 at 37 °C was monitored by applying the HPLC method. The rate and time of the release of biochanin A from PLA fibers is in correlation with the amount of the active ingredient in the matrix of the carrier and follows zero-order kinetics. PLA fibers with biochanin A exhibit concentration-dependent activity on proliferation and migration of L929 fibroblasts in direct culture system in vitro, and proved to be suitable for a potential formulation for use in wound healing.

## 1. Introduction

The design and fabrication of a controlled drug delivery system plays a primary role in reducing dosing frequency and increasing drug efficacy [1]. The fabrication of such an ideal drug delivery system is still unattainable; however, the research in recent years has focused on a number of new materials and techniques for the fabrication of carriers with optimal properties [2,3].

The use of a conventional therapy, most commonly of oral dosage forms, leads to unpredictable absorption and high fluctuations of the drug concentration in the blood [4], which is a serious problem with drugs with a narrow therapeutic range. Controlled drug delivery systems should enhance the stability and absorption of the active ingredient, so that the constant therapeutic drug concentrations can be retained within the target site for as long as possible in order to have a long elimination half-life and to be biocompatible [5]. An ideal carrier should provide the release of pharmacologically active ingredients by means of zero-order kinetics [6,7,8].

Also, carriers of active ingredients must have such properties that they can ensure the preservation of the physical and chemical drug stability and prolong its shelf life. However, after local or systemic drug administration, in addition to the need to enable the controlled release of the active ingredients, it is important that they are degraded in the body within an appropriate period of time without any consequences for human health. Finding new methods and combinations of different compounds for fabrication of a carrier which would be as close to an ideal drug delivery system as possible is a great challenge for researchers.

The electrospinning technique is a simple method based on electrostatic forces that can be used to make fibers from different materials. The diameter of these continuous fibers ranges from a few nanometers to a few micrometers, which is achieved by changing various process parameters. The polymer fibers obtained by the electrospinning technique can also be used as carriers of pharmacologically active ingredients. It is because of the capacity to make very porous nets with a large ratio of specific surface area and volume that they have attracted significant attention of researchers in this field [9,10]. Various drug substances, ranging from antibiotics and cytostatics to proteins and DNA molecules, can be incorporated into carriers with specific characteristics, thus increasing their bioavailability, as well as controlled and targeted release [11,12]. The above-mentioned technique is considered economical and easy to use [13].

Polylactide polymer (PLA) is obtained through polymerization of lactic acid monomer formed through fermentation of sucrose from sugar cane molasses. The presence of two chiral centers in the lactic acid molecule accounts for an opportunity for modification of the physical and chemical properties of PLA [14]. The possibility of adaptation of mechanical, physical, microstructural, chemical and degradation properties for specific applications has triggered extensive research with the aim of using these materials in innovative ways. PLA exhibits very good characteristics such as biocompatibility, biodegradability (hydrolysis and enzyme activity) and low immunogenicity, and as such is one of the basic biomaterials for a number of applications in medicine, pharmacy and industry [15,16,17]. Due to their excellent biocompatibility and mechanical properties, PLA and its copolymers are used in tissue engineering to restore the function of damaged tissues. Also, PLA is widely used in the development of various types of drug delivery systems such as particles, liposomes, dendrimers and micelles, in which different types of drugs can be encapsulated, including cytostatics.

A study conducted by Cui et al. examines the possibility of using PLA polymer as a delivery system for an antimicrobial agent, doxycycline, fabricated by a simple electrospinning technique [18]. Controlled and prolonged release can be achieved by adjusting the content of the active ingredient in the fabricated fibers of a polymer. An advantage of such a drug delivery system in wound healing is its capacity to absorb exudates, which is especially important in the treatment of moisturizing wounds. The studies have shown that PLA foils containing 20% doxycycline actually have the highest capacity of water absorption, as they can absorb an amount of water that is 12.4–17.3 times as large as the mass of the foil itself. Also, PLA foils allow the passage of water vapor, thus allowing wounds to ‘breathe’, although this capacity of the foil decreases with an increase in the drug content in the polymer. The MTT test (test with 3-(4,5-Dimethylthiazol-2-yl)-2,5-diphenyl-2H-tetrazolium bromide) showed no cytotoxic effect, due to the gradual and long-term release of an antimicrobial agent (about two weeks), while its antimicrobial activity was preserved after two weeks, which indicates the preservation of physical and chemical stability of molecules by fitting into this type of carrier. In vivo tests have confirmed an advantage in the treatment of chronic wounds in rats with type 1 diabetes compared to conventional topical preparations for local administration [18].

Electrospun pH sensitive cellulose acetate phthalate fibers are suitable for the delivery of active ingredients in the small intestine, whereas they do not dissolve in the stomach, vaginal environment or in tissue engineering [19,20,21,22]. PLA is suitable for application as a carrier of active ingredients since it is biodegradable and can be implanted in almost any part of the human body since it is non-toxic and because the product made by its degradation, lactic acid, is non-toxic as well. It is easily sterilized and obtained from renewable raw materials.

Also, the influence of the content of an active ingredient and the diameter of the fibers obtained by the electrospinning method on the drug release profile was investigated. Cui et al. have developed PDLA fibers with a diameter of 400 nm with 2%, 5% and 8% paracetamol as well as fibers of different diameters of 212 nm, 551 nm and 1.3 μm with 5% paracetamol [23]. Each example showed a two-phase release (initially fast and later slow), but a constant release of the drug for up to two weeks.

The release of the active ingredient from PLA fibers fabricated from polymer solutions and melts demonstrates certain differences. The release of the pharmacologically active ingredient of chloramphenicol was monitored under simulated physiological conditions (37 °C, pH range 7.2–7.4), whereupon a delay was observed in the onset of rapid release of 1 h from PLA fibers fabricated from polymer melts. The drug release mostly corresponds to Higuchi’s mathematical model of substance release. With a decrease in the diameter of the fibers and an increase in the drug content in the polymer, a higher percentage of drug release in the first rapid phase, as well as in drug release at a higher rate, is observed [24].

The study by Liu et al. examines the effect of asymmetric multilayer films made from PLA fibers by using the electrospinning method in which the antineoplastics oxaliplatin and cyclophosphamide were incorporated on the occurrence of recidives of hepatocellular cancer in vitro and in vivo [25].

Biochanin A, 5,7-dihydroxy-4’-methoxy-isoflavone, belongs to the group of phytoestrogens found primarily in red clover [26] and other plant species belonging to the Fabaceae family [27,28]. The chemical structure of biochanin A shows a similarity with the chemical structure of the female sex hormone, 17-β-estradiol. The presence of a phenolic ring and the corresponding distance between the two oxygens in the structure of this molecule enable its unhindered binding to hydrogen bonds and p-p interactions with the estrogen receptors ER-α and ER-β [29,30,31].

The interaction of biochanin A with estrogen receptors can achieve estrogenic and antiestrogenic effects depending on the endogenous estrogen concentration, the applied dose of biochanin A, the duration of therapy, the affinity to receptor binding, bioavailability, and the health condition and ethnicity of consumers, which is why they are ranked among the selective estrogen receptor modulators (SERMs) [29,32].

Also, the findings of a number of studies indicate that the mechanism of action of biochanin A does not depend only on its interaction with estrogen receptors, but that certain pharmacological activities are actually achieved by binding to a number of other receptor types (PPARγ, PPARα, AhR), as well as by modification of various signalling pathways. κB, MAPK, PI3K/Act) [26,27,33,34,35].

Topically applied Biochanin A can reduce UVB-induced inflammatory processes by directly inhibiting MLK3 kinase in order to prevent photoaging and facilitate wound healing [36,37]. Also, due to the impact on the secretion reduction of IL-8, IL-6 and VEGF, it finds its local application in the treatment of canker sores [38]. The study, by Lin et al. examines the inhibitory effect of biochanin A on the process of melanogenesis in an in vitro and in vivo study, and as such can be used to treat skin hyperpigmentation [39]. For the indication of androgenic alopecia, the local application of a combination of plant extracts containing biochanin A was investigated in comparison to minoxidil 3%, thus leading to the conclusion that the applied phytopreparation may be its adequate therapeutic alternative [40].

A large number of studies have shown that biochanin A has gastroprotective [41], hepatoprotective [42], cardioprotective [43,44], neuroprotective [45], anticancer [26] and antidiabetic activity [46].

The importance of biochanin A is reflected in its potential application in the field of toxicology, as this can lead to a significant improvements in histological changes and intoxication parameters [47,48,49]. However, all the health benefits of biochanin A are limited by its poor water solubility, high clearance and low bioavailability (<4%) [50]. In order to address the aforementioned weaknesses of biochanin A, different formulations have been presented in the reference sources: inclusion complex with cyclodextrins [51], gel type matrix systems [52], and formulations for buccal administration [53]. We have not found any information about electrospun polyactide microfibers with biochanin A in the available reference sources.

The objective of this work is to develop a formulation based on PLA fibers with a different biochanin A content by the using electrospinning method in order to perform characterization and examine the kinetics of release and its effect on wound healing and the proliferation of L929 fibroblasts in in vitro conditions. The formulation of biochanin A with electrospun microfibers can be applied in various pharamceutical forms for systemic and local administration.

## 2. Materials and Methods

### 2.1. Reagents

Biochanin A (BCA), purity of 98% (Sigma Aldrich, Steinheim, Germany); potassium bromide (KBr) for IR spectroscopy, ≤100% (Merck KGaA, Darmstadt, Germany); Hanks’ buffered solution pH 7.4 GmbH (PAA Laboratories, Pasching, Austria); 2-propanol, purity of 99.5% (Centrohem, Belgrade, Serbia) and 3-(4,5-Dimethylthiazol-2-yl)-2,5-diphenyl-2H-tetrazolium bromide (MTT), purity of ≥97.5% (Sigma Aldrich, Steinheim, Germany) were used. Semi-crystalline PLA (consisting of D, L-lactic acid units with low L-lactide content, presumably about 10%), purity of 99% provided from Shenzhen Esun Industrial Co., Ltd. (Shenzhen, China), characterized by a number-average molecular weight (M_n_) of 60.520 g/mol, a weight-average molecular weight (M_w_) of 160.780 g/mol, and polydispersity index (PDI) of 2.66, was used for the preparation of electrospun polymer fibrous matrices. Chloroform, purity of ≥99.5% (Lachner, Neratovice, Czech Republic) and dimethylformamide (Fischer Scientific, Waltham, USA), purity of ≥99.8% were used for the preparation of polymer-based solutions for electrospinning. All chemicals were used as received.

### 2.2. Preparation of Solutions for Electrospinning

For electrospinning, PLA-based solutions were prepared by dissolving appropriate amount of PLA in the mixture of chloroform and dimethylformamide in the volumetric ratio 6:4 (V:V), so that the final concentration of polymer was 9 wt% by a method published earlier [54,55,56]. Active materials were prepared by adding of 2% and 5% of biochanin A (calculated on the polymer weight) to basic polymer solutions. All solutions were mixed 24 h prior to electrospinning on magnetic stirrer at room temperature. Viscosity was measured on MYR viscometer ver V0 model 3000 which is in accordance with ISO 2555/ASTM method. Production of fiber cariers was done on electrospinning machine Fluidnatek LE-10 (manufacturer Bioinicia, Paterna, Spain) and process parameters were adjusted for each prepared solution. Viscosity was measured on MYR viscometer ver V0 model 3000 which is in accordance with ISO 2555/ASTM method. The list of samples with electrospinning process parameters is presented in Table 1.

### 2.3. Stretching of the Material

The mechanical properties of the prepared samples were examined using a tensile testing machine EZ-LX Test (Shimadzu, Kyoto, Japan). The obtained materials were cut in rectangular shaped strips, thickness and width were measured, and the samples were stretched with load of 1 mm/min. Stress (N/mm^2^) and stroke-strain (%) were followed in maximum and break point.

### 2.4. Determination of the Wetting Angle

Surface properties were determined using a Contact Angle Goniometer (Ossila, Sheffield, UK) with water as a wetting medium. The drop (5 μL) was dripped onto the surface of material and contact angle was measured.

### 2.5. Differential Scanning Calorimetry (DSC)

Differential scanning calorimetry (DSC) was used for the examination of thermal properties of the obtained materials. A small amount of sample (5 mg) was put into a pan and heated in one cycle from room temperature to 250 °C at the speed of 10 °C/min in a nitrogen atmosphere. The TA Instruments Q20 differential scanning calorimeter (TA Instruments, New Castle, DE, USA) was used for these tests.

### 2.6. Fourier Transform Jnfrared Spectroscopy (FTIR)

The electrospun PLA fibers with and without biochanin A were ground to powder in an amalgamator (WIG-L-BVG, 31210-3A, Dentsply RINN, a Division of Dentsply International Inc., York, PA, USA). FTIR spectra of the biochanin A, electrospun PLA fibers, PLA-BCA-2% and PLA-BCA-5% were recorded using the technique of thin transparent pastilles, by vacuuming and pressing under the pressure of about 200 MPa. The pastilles were prepared by mixing 150 mg of KBr and 0.7 mg of the sample. FTIR spectra were recorded in the wavenumber range of 4000–400 cm^−1^ on a Bomem Hartmann & Braun MB-series FTIR spectrophotometer (Bomem Hartmann & Braun, Quebec, QC, Canada). The obtained spectra were analyzed using the Win-Bomem Easy software.

### 2.7. Scanning Electron Microscopy (SEM)

Scanning electron microscopy (SEM) was used to examine the morphology of the electrospun PLA fibers with and without biochanin A. The samples were sprayed by an alloy of gold and palladium (85%/15%) under vacuum in a Fine Coat JEOL JFC-1100 Ion Sputter (JEOL Ltd., Tokyo, Japan). The metalized samples of electrospun PLA fibers were scanned using a JEOL Scanning Electron Microscope JSM-5300 (JEOL Ltd., Tokyo, Japan), under a magnification of 10,000 times, voltage 20 kV, vacuum 1.33 × 10^−5^ Pa.

### 2.8. Modified Release of Biochanin A from Electrospun PLA Fibers

The samples of electrospun PLA fibers (9 mg) with 2% and 5% of biochanin A were soaked in with 10 cm^3^ of Hanks’ buffer (pH = 7.4). The samples were stirred (120 rpm) and thermostated in a water bath at 37 °C. The release of biochanin A was monitored by sampling 200 µL of the solution at certain time intervals and diluting with 800 µL of methanol. All samples were filtered on the Econofilter with the pore diameter of 0.45 μm and analyzed by using the HPLC method. Simultaneously, dissolution of biochanin A in Hanks’ buffer was examined under the same conditions by suspending 0.46 mg of biochanin A in 10 cm^3^ of Hanks’ buffer. For the construction of the calibration curve, a series of standard solutions of biochanin A in methanol (1–100 μg/cm^3^) was prepared. The dependency of peak area on biochanin A concentration is linear, with a correlation coefficient R^2^ = 0.999, and is represented by Equation (1):

This is example 1 of an equation:

(1) A=30.06+129.53·C

where C (μg/cm^3^) is the concentration of biochanin A.

### 2.9. High Performance Liquid Chromatography (HPLC)

The high performance liquid chromatography (HPLC) method was applied for the quantitative analysis of biochanin A released from electrospun PLA fibers, as well as for solubility testing of biochanin A in Hanks’ buffer. A mobile phase (800 μL) was added to every sample (200 μL) taken at a certain time interval. All samples were filtered on the Econofilter with the pore diameter of 0.45 μm and analyzed on Agilent Technologies 1100 Series HPLC device under the following conditions: column: ZORBAX Eclipse XDB-C18 (4.6 × 250 mm, 5 μm); mobile phase: methanol; flow rate: 1 cm^3^/min; detection: DAD detector Agilent Technologies 1200 Series, λ = 265 nm; temperature: 30 °C; injected sample volume: 20 μL.

### 2.10. Cell Proliferation Assay

The L929 fibroblast cell line (mouse skin fibroblasts) was used for in vitro studies of wound healing activity of electrospun PLA fibers with and without biochanin A. L929 fibroblasts were cultivated in Dulbecco’s Modified Eagle Medium (DMEM) containing 10% fetal bovine serum (FBS), 2 mM stable glutamine, and antibiotic-antimycotic solution (complete DMEM), at 37 °C in a humidified environment containing 5% CO_2_. All cell culture reagents were purchased from Capricorn Scientific GmbH, Germany.

For the cell proliferation assay, L929 cells were seeded in standard 24 well plates (Greiner Bio-One, Germany) at a density of 1 × 10^4^ cells per well. Twenty-four hours after the cultivation of cells, samples of electrospun PLA fibers were added to the cells (direct contact assay). The dimensions of the tested samples were 1 cm × 1 cm. The cells incubated only with the medium without the tested material (complete DMEM) were used as a control cell culture (untreated cells). Each tested sample was examined in three replicates, and so was the control culture. The cells were incubated with the tested samples or control medium for the next 72 h. After the incubation period ended, an MTT test was performed according to the previously established protocol [57].

The MTT test is widely used for assessment of cell proliferation and is based on the reduction of 3-(4,5-dimethylthiazol-2-yl)-2,5-diphenyl-2H-tetrazolium bromide (tetrazolium salt MTT) by mitochondrial dehydrogenases of living cells, resulting in formazan crystals formation that corresponds to the number of cells. The cells were washed with phosphate buffer saline and then 300 μL of MTT solution per well (concentration 1 mg/mL) was added to the cells. The cells were incubated with MTT solution for the next three hours followed by formazan crystals dissolution with 2-propanol. The absorbance of dissolved formazan was measured on a Multiskan Ascent Photometric plate reader (Thermo Labsystems, Helsinki, Finland) at a wavelength of 540 nm with correction wavelength of 650 nm. The mean absorbance values were calculated for each tested sample, as well as for the control cell culture. The cell proliferation rate was calculated according to the following formula:

% cell proliferation = (absorbance value of cells treated with fibers/absorbance value of untreated cells) × 100(2)

### 2.11. In Vitro Wound Healing Assay

To examine the effects of electrospun PLA fibers without and with 2 and 5% biochanin A on wound healing in vitro, we performed a ‘scratch’ assay according to our previously published protocol [58,59]. Briefly, L929 fibroblasts were seeded in a sterile 48 well plates and incubated under the standard cell culture conditions (37 °C, 5% CO_2_ and in humidified environment). After reaching the 100% confluence, a wound (‘scratch’) was created in a cell monolayer, in the middle of each well. The cells were then washed with buffer and samples of electrospun PLA fibers with and without biochanin A, or complete DMEM, were added. Each sample, as well as the control one, was tested in three replicates and the experiment was performed twice under the same conditions. The fibroblasts were incubated with the samples of electrospun PLA fibers without and with 2 and 5% biochanin A and the effect on wound’s closure was monitored after three days of incubation. A microscopic analysis of the wound’ closure was performed on an inverted light microscope, an Axio Observer.Z1 (Carl Zeiss, Oberkochen, Germany), and morphometric measurements were made in ZEN 2 (blue edition) software (Carl Zeiss, Oberkochen, Germany) after imaging. The extent of wound closure was determined by measuring the width of the wound area before incubation with electrospun PLA fibers with and without biochanin A and three days after the incubation with the samples as well as with complete medium (comtrol), and is expressed as the percentage of wound closure.

### 2.12. Statistical Analysis

The results of the MTT proliferation assay as well as the in vitro wound healing assay of at least two independent experiments were analyzed using one-way analysis of variance (ANOVA). MTT test results were expressed as a percentage of cell proliferation with relative standard deviation calculated according to the control culture of cells for which the cell proliferation rate was considered to be 100%. As statistically significant differences, we considered those for which *p* < 0.05. The data analysis was performed using the software package SPSS Statistics version 20.0 (IBM, Chicago, IL, USA).

## 3. Results

### 3.1. Preparation and Characterization of Electrospun PLA Fibers with and without Biochanin A

**Figure 1:**
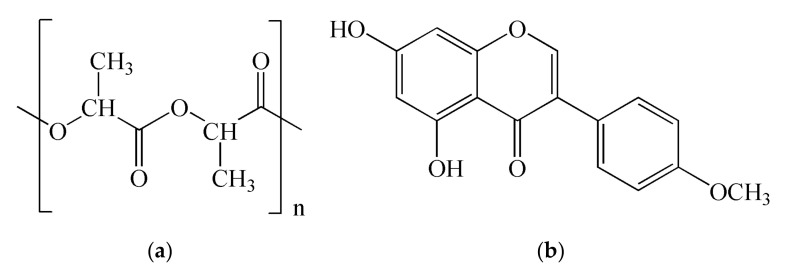
Structure of (**a**) poly(lactide) and (**b**) biochanin A.

Electrospun PLA fibers were prepared using 9 wt% solution of PLA in a chloroform dimethylformamide mixture (6:4, V/V). The structure of poly(lactide) is shown in Figure 1a.

This combination of solvents enables satisfactory volatility of the solvent from the polymer solutions during the electrospinning process. In addition to that, the prepared solutions have an appropriate viscosity of (400 mPa⋅s), which is a very important parameter for the morphology of obtained fibers.

The fibers with biochanin A were prepared by electrospinning of PLA polymer solutions with 2% or 5% of biochanin. The structure of biochanin A is shown in Figure 1b.

The electrospinning process is performed at a room temperature which is very important for thermosensitive active substances, such as biochanin A, because thermal degradation of biochanin A is prevented and its stability is maintained.

#### 3.1.1. Mechanical Properties of Electrospun PLA Fibers

Mechanical properties of the samples are summarized in Table 2. Measuring was conducted on five samples and the mean value was taken. When comparing the mechanical properties of the samples within a PLA-based series of materials, it can be concluded that the presence of biochanin A induced a slight increase of maximum stress, while elongation was significantly higher for the samples with 5% of biochanin A. The elongation of the material at the point of maximum stress was lower for the samples with higher biochanin A content: 4.69% for pure PLA and 4.00 for PLA-BCA-5%, which is not a significant decrease.

Presented results showed that the mechanical properties of PLA have not changed significantly in the presence of biochanin A and that electrospun PLA fibers can be used as a carrier in formulations for controlled delivery of biochanin A.

#### 3.1.2. Surface Properties of Electrospun PLA Fibers

Table 3 shows the values of the contact angle of the samples of electrospun PLA fibers with and without biochanin A. The hydrophobic properties of biochanin A affected the surface properties of the obtained active materials compared to pure polymer materials. Pure electrospun PLA fibers already have hydrophobic properties, and the addition of 2% of biochanin A increased the contact angle by 17°, while 5% of biochanin A increased the contact angle by another 8°. This may indicate that the PLA fibers have been saturated with BCA molecules on the surface so that any further increase in the BCA concentration in PLA has less of an effect on hydrophobicity and the contact angle.

#### 3.1.3. Thermal Properties of Electrospun PLA Fibers

**Figure 2:**
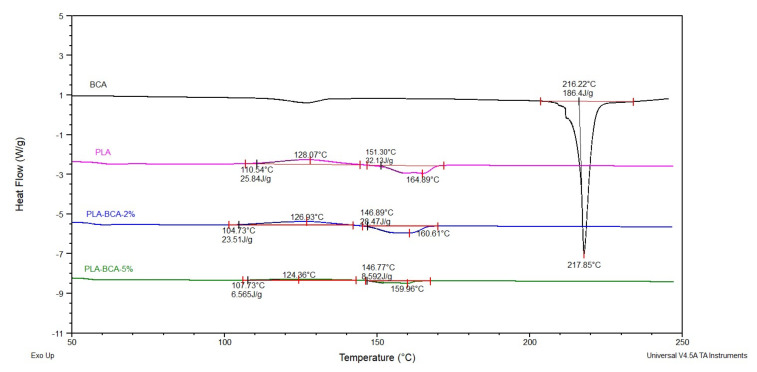
DSC curves of: biochanin A, electrospun PLA fibers, PLA-BCA-2% and PLA-BCA-5%.

The results of the DSC analysis are shown in Figure 2. Due to good compatibility of PLA matrix and biochanin A, which was confirmed by the results of the analysis of surface and mechanical properties, satisfactory incorporation of biochanin A into the electrospun fibers can be expected. Due to complete incorporation of BCAinto the fibers, the DSC curves of electrospun fibers with BCAdo not contain peaks corresponding to the melting temperature of BCA at 216 °C. This incidence is according to the reference sources, e.g., absence of the melting peak of chlorhexidine in the membrane of cellulose acetate phthalate fibers [22].

DSC curves of PLA-BCA-2% and PLA-BCA-5% showed thermal transitions characteristic for PLA, such as a T_g_ value of about 60 °C, cold crystallization, and melting. The maximum crystallization temperature is decreased from 128.07 °C for PLA, to 126.93 °C for PLA-BCA-2% and 124.36 °C for PLA-BCA-5%. The enthalpy of crystallization is also decreased from 25.84 J/g for PLA, to 23.51 J/g for PLA-BCA-2% and 6.56 J/g for PLA-BCA-5%. The melting temperature of PLA is reduced from 164.89 °C to 160.61 °C for PLA-BCA-2% and 159.96 °C for PLA-BCA-5%. The enthalpy of melting is also reduced from 32.13 J/g for PLA, to 28.47 J/g for PLA-BCA-2% and 8.59 J/g for PLA-BCA-5%. The lower temperature of cold crystallization and enthalpy for higher concentration of biochanin A are the result of the nucleating effects or acceleration of biochanin A crystallization, whereupon the lower cold crystallization temperature induces a lower melting temperature.

Based on all of the above, it can be concluded that the incorporation of biochanin A into a PLA matrix moderately affects its thermal properties, with a more pronounced effect on the enthalpy.

#### 3.1.4. Morphology of the Electrospun PLA Fibers

**Figure 3:**
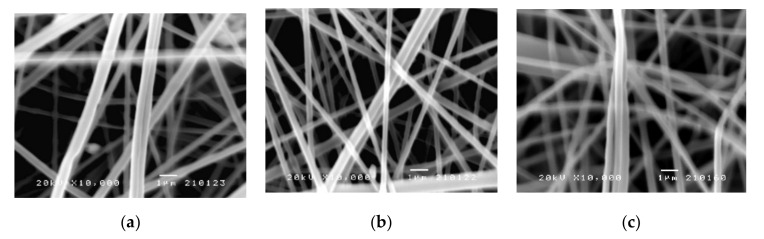
SEM images of electrospun fibers of: (**a**) PLA, (**b**) PLA-BCA-2%, and (**c**) PLA-BCA-5% (‘bar’ 1 μm; magnification 10,000×).

The morphology of the electrospun PLA fibers was examined by using scanning electron microscopy (SEM). The SEM images of PLA fibers with and without biochanin A are shown in Figure 3. The electrospun PLA fibers were round and smooth (Figure 3a) without any visible deformations and about 1 μm thick. The addition of biochanin A did not affect appearance of fibers (Figure 3b,c) and neither crystals nor aggregates of the active ingredient can be noticed. This means that biochanin A is completely incorporated into the polymer fibers and that parameters of electrospinning process are well defined. The small diameter of the obtained PLA fibers gives them a large specific surface area and shorter diffusion route, which is very important for the delivery of poorly soluble active substances such as biochanin A. In addition, a large specific surface area can facilitate mass transfer and enable the efficient release of biochanin A.

#### 3.1.5. FTIR Analysis

**Figure 4:**
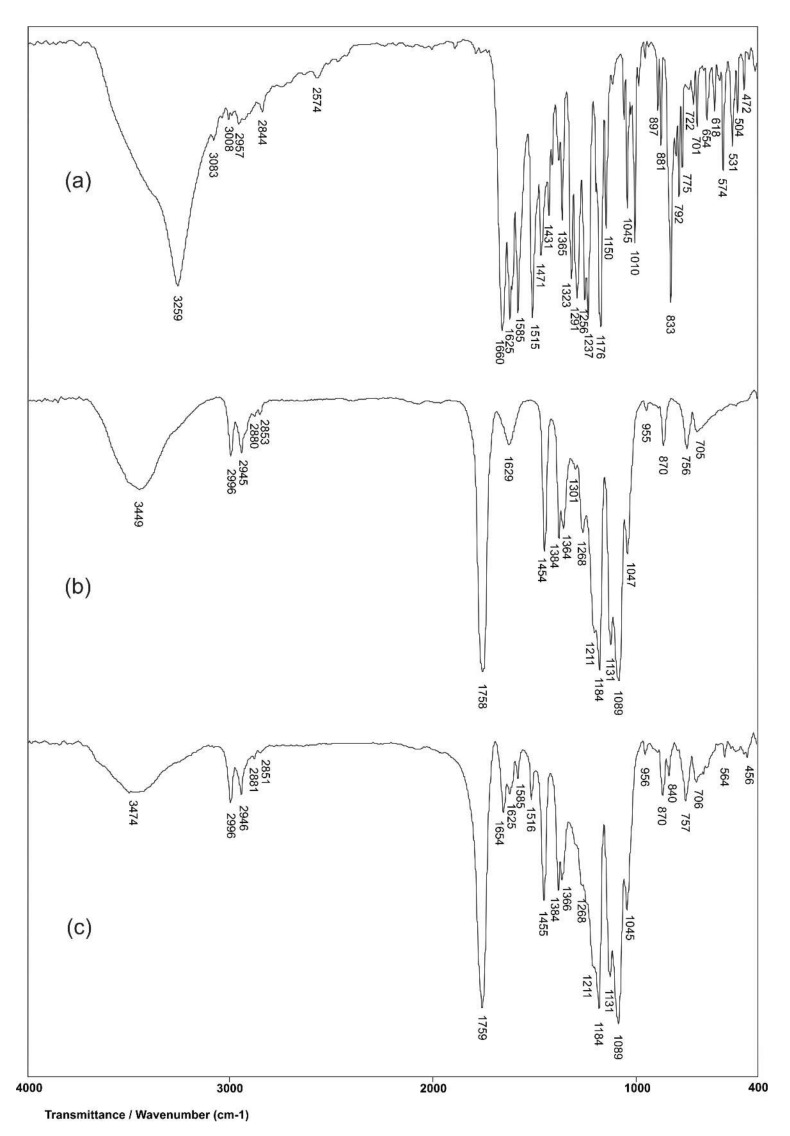
FTIR spectra of: (**a**) biochanin A, (**b**) PLA and (**c**) electrospun PLA-BCA-5% fibers.

The structural characterization of biochanin A, PLA, and PLA-BCA-5% is performed by using the FTIR method. In the FTIR spectrum of biochanin A (Figure 4a), wide, intensive absorption band with maximum at 3259 cm^−1^ originates from valence vibrations of phenolic OH groups, ν(OH). Characteristic valence vibrations of the phenolic C–O bond, ν(C–O)Ar, give intensive bands in the range of 1260–1000 cm^−1^, and this band is present in the spectrum of biochanin A at 1176 cm^−1^. In-plane deformation vibrations of hydroxyl groups, δ(OH), give a low-intensity band with a maximum at 1323 cm^−1^. The strong absorption band at 1661 cm^−1^ is a result of valence vibrations of the carbonyl group, ν(C=O). The characteristic absorption bands at 1625, 1585 and 1515 cm^−1^ originate from valence vibrations of aromatic double bonds, ν(C=C)Ar,. The asymmetric valence vibrations of ether C–O–C bond, ν_as_(C–O–C), give two bands at 1258 and 1237 cm^−1^.

In the FTIR spectrum of PLA (Figure 4b), two bands are present, with maxima at 2853 and 2945 cm^−1^, respectively, originating from symmetric and asymmetric valence vibrations of C–H bonds that are present. Symmetric and asymmetric valence vibrations of C-H bonds from the CH_3_ group give two bands at 2880 and 2996 cm^−1^. A wide absorption band originating from valence vibrations of the hydroxyl group is present in the range of 3200–3600 cm^−1^, and this band has maximum at 3449 cm^−1^ in the spectrum of PLA. Symmetric and asymmetric valence vibrations of C–O–C group give two bands with maxima at 1089 and 1184 cm^−1^, respectively. The absorption band at 1758 cm^−1^ originates from valence vibrations of the aliphatic ester C=O group. The bands at 1454 and 1394 cm^−1^ are usually attributed to symmetric and asymmetric deformation vibrations of C-H bonds in methyl groups, respectively. Also, the overlapping of bending vibrations of C-H bonds with stretching vibrations of C–O–C bonds gives the band at 1268 cm^−1^ in the spectrum of PLA.

In order to examine interactions between biochanin A and PLA, FTIR spectra of PLA with and without biochanin A were analyzed and vibration frequencies of the significant groups are shown in Table 4.

**Figure 5:**
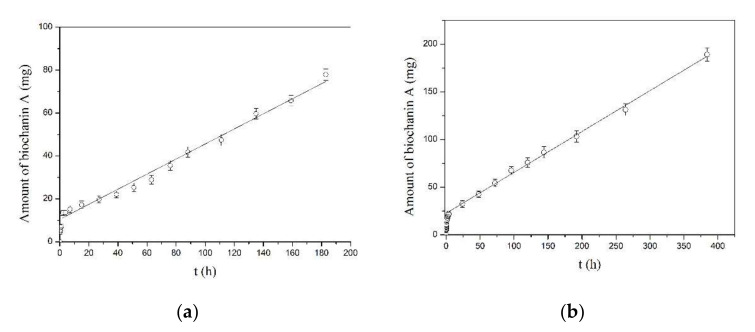
Amount of biochanin A released from (**a**) electrospun PLA-BCA-2% and (**b**) electrospun PLA-BCA-5% fibers in buffer at pH 7.4 and temperature of 37 °C.

By comparing the values of the vibration frequencies in the FTIR spectra of PLA and PLA-BCA-5%, it can be observed that there are no significant differences (0–2 cm^−1^). The results of this analysis showed that the mentioned groups from PLA do not participate in the formation of any intermolecular bonds with biochanin A. Also, valence vibrations of aromatic double bonds, ν(C–O)Ar, at 1625, 1585 and 1515 cm^−1^ in the spectrum of biochanin A (Figure 5a) do not change their positions in the spectrum of PLA-BCA-5% (Figure 4c), which indicates that biochanin A is present in the polymer but does not participate in the intermolecular bond formation. This is very convenient, because it allows biochanin A release from electrospun PLA fibers by diffusion.

### 3.2. Modified Release of Biochanin A from Electrospun PLA Fibers

The amount of biochanin A released over time from PLA-BCA-2% and PLA-BCA-5% in Hanks’ buffered solution pH 7.4 during time is shown in Figure 5a,b, respectively. The results show that amount of released biochanin A increases over time during time at a constant release rate. The release rates of biochanin A from PLA-BCA-2% and PLA-BCA-5% can be calculated as the slope of the straight line and their values were 0.3511 μg/h and 0.4287 μg/h, respectively. The total release time of biochanin A was 23 days for PLA-BCA-2% and 42 days for PLA-BCA-5%.

In order to get a better insight into the biochanin A release kinetic, obtained experimental data were fitted to the appropriate mathematical models (zero order, first order, Korsmeyer-Peppas, Baker-Lonsdale, Higuchi and Makoid-Banakar) using DDSolver, and calculated parameters are shown in Table 5.

The mathematical models used to fit dissolution data facilitate the analysis and interpretation of the obtained results based on several parameters that can be compared statistically. In this study, the appropriate model is selected based on the values of the following parameters: adjusted coefficient of determination (R^2^_adj._), Akaike information criterion (AIC) and model selection criterion (MSC). The adequate model should have high R^2^_adj._ and MSC values, and low AIC value. The model that best describes the in vitro dissolution of biochanin A from electrospun PLA fibers is the Makoid-Banakar model (R^2^_adj._ = 0.9912 for PLA-BCA-2% and 0.9976 for PLA-BCA-5%). In addition, this model has the lowest AIC values (60.45 for PLA-BCA-2% and 33.88 for PLA-BCA-5%) and the highest MSC values (4.44 for PLA-BCA-2% and 5.77 for PLA-BCA-5%), compared to other applied models. The obtained results are in accordance with the result of other authors who monitored the release of active substances from electrospun fibers [63].

The effect of the initial release of biochanin A from PLA-BCA-2% and PLA-BCA-5% fibers is almost completely avoided, while this is a common undesirable phenomenon in some other carriers, such as liposomes, microspheres and microcapsules. The further release of biochanin A is constant and stable and follows zero order kinetic (Table 6), which implies that biochanin A is uniformly distributed in the electrospun fibers.

**Figure 6:**
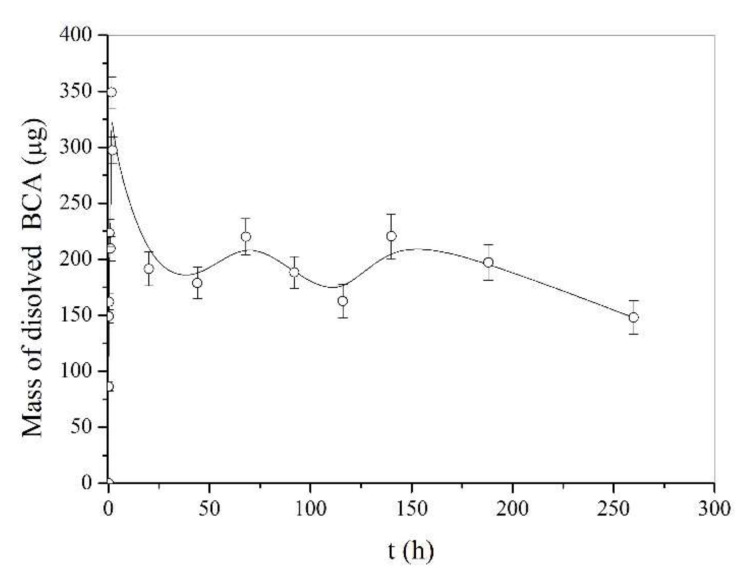
Behavior of biochanin A in a buffer solution pH 7.4 at 37 °C.

On the other hand, the release profile of the active substance depends on the proper polymer selection, which should be in accordance with the nature of the active ingredient. Lipophilic polymers should be used for lipophilic drugs and hydrophilic polymers should be used for hydrophilic drugs in order to achieve an adequate drug release profile. The lipophilic nature of biochanin A is in accordance with the characteristics of PLA as a chosen carrier for the preparation of electrospun fibers, which led to a controlled release of biochanin A at a constant rate. The behavior of pure biochanin A in buffer solution at a pH 7.4 is very unsteady (Figure 6), which confirms the importance of the prepared formulation, because it provides controlled and prolonged release of biochanin A and that contributes to its safe and effective application.

### 3.3. Biological Testing

#### 3.3.1. Cell Proliferation

**Figure 7:**
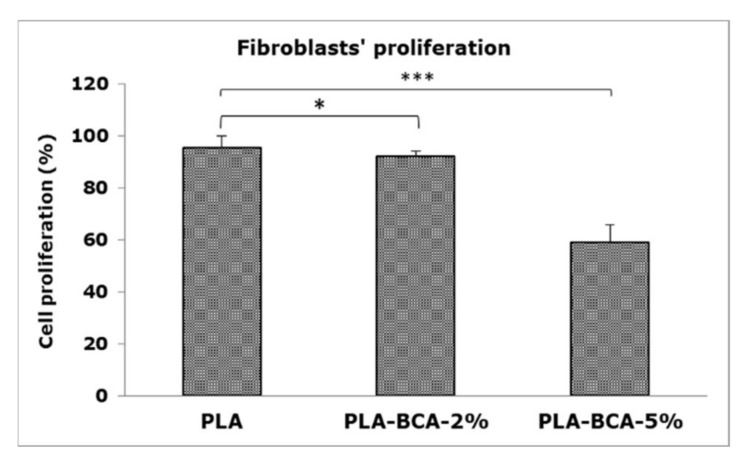
Results of MTT test showing the effect of examined electrospun PLA fibers with 2% and 5% BCA and without BCA on proliferation of L929 fibroblasts; (*) *p* < 0.05, (***) *p* < 0.001.

The effects of electrospun PLA fibers with biochanin A (PLA-BCA-2% and PLA-BCA-5%) and without biochanin A (PLA) on fibroblasts’ proliferation are shown in Figure 7.

The concentration-dependent effect of biochanin A released from PLA fibers on the proliferation of L929 fibroblasts was noticed in the used direct culture system. Cell proliferation was slightly decreased in PLA-BCA-2% (92%) and moderately decreased in the PLA-BCA-5% (59%) group, both statistically significantly different from the control (untreated cells) and pure PLA.

**Figure 8:**
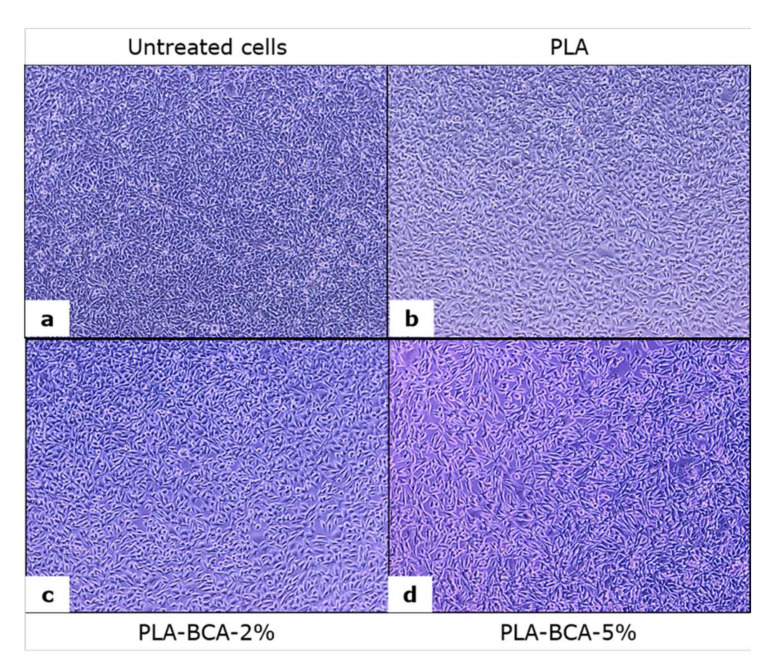
L929 cells after three days of incubation with standard cell culture medium (untreated cells) without electrospun fibers (**a**), with PLA (**b**), PLA-BCA-2% (**c**), and PLA-BCA-5% (**d**) electrospun fibers.

Changes in L929 cell number and morphology under the influence of examined samples of electrospun PLA fibers that can be observed in Figure 8 corresponds with the results of the MTT test. Particularly, the cells incubated with PLA-BCA-5% (Figure 8d) were larger in size, elongated and with noticeable cell extensions. The changes in morphology are in accordance with the released amount of biochanin A and were more pronounced in the PLA-BCA-5% compared to the other groups. Due to poor PLA solubility in the cell culture medium, biochanin A was gradually released from electrospun PLA-BCA-2% and PLA-BCA-5% fibers and the effect on cells depends on the released biochanin A concentration.

#### 3.3.2. In Vitro Wound Healing Activity

In vitro wound healing activity was examined using the in vitro “scratch” assay. The wound (scratch) was created in a confluent cell monolayer which was followed by incubation with the electrospun fibers or complete medium (control).

**Figure 9:**
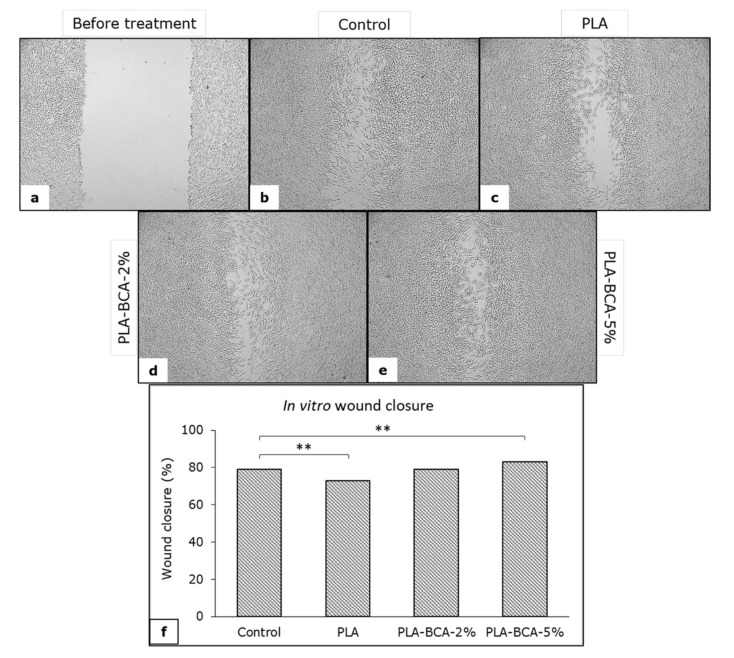
Appearance of in vitro created “wounds” before (**a**) and three days after incubation with complete medium (control) (**b**), PLA (**c**), PLA-BCA-2% (**d**) and PLA-BCA-5% (**e**); as well as percentage of wound closure (**f**); (**) *p* < 0.01.

The results of the in vitro wound healing activity of electrospun PLA fibers, as well as the appearance of the ‘wounds’ after three days of incubation are presented in Figure 9. The greatest extent of ‘wound’ closure was achieved with PLA-BCA-5% electrospun fibers and the lowest with electrospun PLA fibers without biochanin A, both significantly different from control (*p* < 0.01).

The electrospun PLA fibers are not soluble in water and represent stable carriers for the controlled release of biochanin A. The observed effects of biochanin A on cell proliferation and in vitro wound healing activity are concentration-dependent. Electrospun PLA-BCA-5% fibers have a moderate antiproliferative effect, but a more stimulating effect on cell migration and wound closure than PLA-BCA-2% and complete medium without fibers. The presented results show that electrospun PLA fibers with 5% of biochanin A can be a potential formulation for use in wound healing. However, in order to further develop a potential formulation, some in vivo analyses are necessary, such as with animal experimentations.

## 4. Conclusions

The introduction of biochanin A into PLA fibers was performed by uniaxial electrospinning, which ensures the uniform dispersion of biochanin A in the polymer matrix. The FTIR analysis of biochanin A, PLA and PLA fiber with biochanin A shows that there are no chemical interactions between the polymer and the active ingredient, which is important for the smooth release of biochanin A. The obtained fibers are of fine structure and without any deformations which they retain even in the presence of biochanin A, which is in correlation with the applied process parameters during electrospinning. The surface properties of fibers change with the addition of biochanin A, become hydrophobic and are closely related to the amount of biochanin A present, while the mechanical properties are slightly changed. The rate and time of the release of biochanin A from PLA fibers depend to a large extent on the concentration of biochanin A in the polymer fiber matrix. The release kinetics, independent of the concentration of biochanin A in the fibers, corresponds to a mathematical model of zero order and the release was prolonged for more than 400 h. PLA fibers with biochanin A exhibit concentration-dependent activity on proliferation and migration of L929 fibroblast cells in a direct culture system in vitro, and proved to be suitable for a potential formulation for use in wound healing.

## 5. Patents

Patent Application RS2022/P0130, Gajić, I.; Stojanović, S.; Ristić, I.; Pilić, B.; Ilić-Stojanović, S.; Nešić, A.; Najman, S.; Dinić, A.; Stanojević., L.; Urošević, M.; Nikolić, V. and Nikolić, L.; Formulations of electrospun polylactide fibers with phytoestrogens for prolonged release, Priority 9 February 2022, the Intellectual Property Office of the Republic of Serbia.

## Figures and Tables

**Table 1:** Samples and process parameters of electrospinning.

Sample Name	Electrospinning Process Parameters		
	Flow Rate (cm^3^/h)	Needle-to-Collector Distance (cm)	Voltage (kV)
PLA	2.5	10	14
PLA-BCA-2%	3	10	13
PLA-BCA-5%	3	10	14

**Table 2:** Mechanical properties of electrospun PLA fibers with and without biochanin A.

Sample Name	Max Stress (N/mm^2^)	Max Stroke-Strain (%)	Break Stress (N/mm^2^)	Break Stroke-Strain (%)
PLA	0.44	4.69	0.53	5.47
PLA-BCA-2%	0.566	4.42	0.22	6.78
PLA-BCA-5%	2.083	4.00	0.35	37.6

**Table 3:** Contact angle of electrospun PLA fibers with and without biochanin A.

Sample Name	Contact Angle (°)
PLA	101.53
PLA-BCA-2%	118.13
PLA-BCA-5%	126.63

**Table 4:** Comparative values of the vibration frequencies in the FTIR spectra of PLA and PLA-BCA-5%.

Vibration	Peak in the Spectrum of PLA, cm^−1^	Peak in the Spectrum of PLA-BCA-5%, cm^−1^
ν_s_(C-H)	2853	2851
ν_as_(C-H)	2945	2946
ν_s_(C-H) from CH_3_	2880	2881
ν_as_(C-H) from CH_3_	2996	2996
ν_s_(C-O-C)	1089	1089
ν_as_(C-O-C)	1184	1184
ν(C=O)	1758	1759

**Table 5:** Applied kinetic models and obtained parameters.

Kinetic Model	Parameter	PLA-BCA-2%	PLA-BCA-5%
**Zero Order** $F=k_{0}\cdot t$	*k*_0_ R^2^_adj._ AIC MSC	0.245 0.9089 100.85 2.19	0.118 0.9282 93.35 2.47
**First Order** $F=100\cdot \left[1-e^{-k_{1}\cdot t}\right]$	*k*_1_ R^2^_adj._ AIC MSC	0.003 0.9237 97.66 2.37	0.001 0.9499 86.87 2.83
**Korsmeyer-Peppas** $F=k_{KP}\cdot t^{n}$	*k_KP_* *n* R^2^_adj._ AIC MSC	1.157 0.679 0.9456 92.48 2.66	0.696 0.678 0.9718 77.44 3.35
**Baker-Lonsdale** $\frac{3}{2}\left[1-{\left(1-\frac{F}{100}\right)}^{2/3}\right]-\frac{F}{100}=k_{BL}\cdot t$	*k_BL_* R^2^_adj._ AIC MSC	0 0.9170 99.17 2.28	0 0.9436 88.99 2.71
**Higuchi** $F=k_{H}\cdot t^{1/2}$	*k_H_* R^2^_adj._ AIC MSC	2.654 0.9285 96.50 2.43	1.779 0.9534 85.54 2.90
**Makoid-Banakar** $F=k_{MB}\cdot t^{n}\cdot e^{-ct}$	*k_MB_* *n* *c* R^2^_adj._ AIC MSC	4.836 0.220 −0.006 0.9912 60.45 4.44	2.905 0.310 −0.002 0.9976 33.88 5.77

R^2^_adj._: adjusted coefficient of determination; AIC: Akaike information criterion; MSC: model selection criterion; *F*: fraction of drug released at time *t*; *k*_0_: zero-order release constant; *k*_1_: first-order release constant; *k_KP_*: release constant in Korsmeyer-Peppas model which incorporating structural and geometric characteristics of the drug dosage form; *n*: Korsmeyer-Peppas release (diffusion) exponent (*n* ≤ 0.43—Fickian diffusion; 0.43 < *n* < 0.85—anomalous behavior, non-Fickian diffusion; *n* ≥ 0.85—zero-order release); *k_BL_*: combined release constant in Baker-Lonsdale model; *k_H_*: Higuchi release constant; *k_MB_*: Makoid-Banakar release constant; *n* and *c*: empirical parameters in Makoid-Banakar model [60,61,62].

**Table 6:** Parameter values obtained for zero-order with F_0_ kinetic model.

Kinetic Model	Parameter	PLA-BCA-2%	PLA-BCA-5%
Zero order with *F*_0_ $F=F_{0}k_{0}\cdot t$	*k*_0_ *F*_0_ R^2^_adj._ AIC MSC	0.195 5.814 0.988 45.66 4.22	0.097 4.694 0.997 21.61 5.48

R^2^_adj._: adjusted coefficient of determination; AIC: Akaike Information Criterion; MSC: Model Selection Criterion; *F*: fraction of drug released at time *t*; *F*_0_: initially released fraction of the drug; *k*_0_: zero-order release constant.

## Data Availability

Not applicable.
